# Dietary Patterns and Obesity in Chinese Adults: A Systematic Review and Meta-Analysis

**DOI:** 10.3390/nu14224911

**Published:** 2022-11-20

**Authors:** Karen Jiang, Zhen Zhang, Lee Ann Fullington, Terry T. Huang, Catherine Kaliszewski, Jingkai Wei, Li Zhao, Shuyuan Huang, Amy Ellithorpe, Shenghui Wu, Xinyin Jiang, Liang Wang

**Affiliations:** 1 Department of Health and Nutrition Sciences, Brooklyn College, City University of New York, Brooklyn, NY 11210, USA; 2 Department of Cardiology, The Affiliated Hospital of Southwest Jiaotong University, The Third People’s Hospital of Chengdu, Cardiovascular Disease Research Institute of Chengdu, Chengdu 610031, China; 3 Library Department, Brooklyn College of the City University of New York, Brooklyn, NY 11210, USA; 4 Center for Systems and Community Design and NYU-CUNY Prevention Research Center, Graduate School of Public Health and Health Policy, City University of New York, New York, NY 10027, USA; 5 Department of Public Health, Robbins College of Health and Human Science, Baylor University, Waco, TX 76798, USA; 6 Department of Epidemiology and Biostatistics, Arnold School of Public Health, University of South Carolina, Columbia, SC 29208, USA; 7 West China School of Public Health, Sichuan University, Chengdu 610017, China; 8 Rory Meyer College of Nursing, New York University, New York, NY 10010, USA; 9 Department of Public Health and Exercise Science, Beaver College of Health Sciences, Appalachian State University, Boone, NC 28608, USA

**Keywords:** dietary pattern, obesity, body weight, Chinese

## Abstract

Certain dietary patterns are associated with an increased risk of obesity and its comorbidities. However, these associations vary across populations. The prevalence of obesity has been rising amid a drastic nutrition transition in China during the country’s rapid economic growth. This systematic review and meta-analysis were conducted to summarize how dietary patterns are associated with obesity in the Chinese population. We searched for articles from 1 January 2000 to 1 February 2022 in PubMed, Cumulative Index to Nursing and Allied Health Literature (CINAHL), and Scopus that assessed the relationship between dietary patterns and obesity outcomes. Odds ratios (ORs) and 95% confidence intervals (CIs) were estimated using a random effects model. From the 2556 articles identified from the search, 23 articles were included in the analysis. We found that the traditional Chinese dietary pattern was associated with a lower risk of overweight/obesity (OR = 0.69, 95% CI: 0.57, 0.84, *p* < 0.001), whereas the Western dietary pattern was associated with a higher OR of overweight/obesity, but not reaching statistical significance (OR = 1.34, 95% CI: 0.98, 1.84, *p* = 0.07). There were inconsistent results for other dietary patterns, such as meat/animal protein and plant/vegetarian patterns. In conclusion, the traditional Chinese diet characterized by vegetables, rice, and meat was associated with a lower risk of obesity. The heterogeneity in characterizing dietary patterns contributes to the inconsistency of how dietary patterns are associated with obesity in the Chinese population.

## 1. Introduction

Obesity, a leading public health problem, has resulted in tremendous medical burdens worldwide [1,2]. It is also a major risk factor for various chronic diseases such as type 2 diabetes, cardiovascular disease, and cancer [3,4,5]. The World Health Organization fact sheet indicates that 39% of adults (≥18 years) are overweight and 13% are obese worldwide [6]. In China, the national prevalence estimates for 2015–2019 were 34.3% for overweight and 16.4% for obesity in adults [7]. Although there are inherent differences in body size and stature across various populations [8], lifestyle factors such as dietary intake that affect energy balance are recognized as important determinants of the current obesity epidemic [9].

A number of foods, nutrients, and dietary components have been associated with weight management and the risk of obesity in observational or interventional studies [10,11,12,13]. However, using the reductionist approach to assess the influence of individual nutrients or food components on body weight cannot fully capture the interaction between dietary components and the complexity of the whole diet [14]. Thus, this may hinder their use for weight management in practice. To overcome such limitations, recent studies have focused on the relationship between a holistic diet (i.e., overall dietary pattern (DP)) and obesity. DP can be derived through an a priori approach using a set of pre-determined criteria (e.g., Healthy Eating Index (HEI) or the Mediterranean Diet Score (MDS)) to characterize dietary intake or through an a posteriori approach using factor analysis of dietary intake data to summarize the nutritional characteristics of a population [15].

An umbrella review of existing systematic reviews regarding DP and obesity demonstrated that the a priori MDS was associated with a reduced risk of obesity in a number of studies [16]. Studies that summarized dietary patterns (DPs) using the a posteriori approach have substantial heterogeneities in pattern identification. A fruit- and vegetable-rich pattern was associated with a reduced risk of obesity [17], whereas a Western pattern characterized by fast food, meat, deep-fried and processed food, and sugar-sweetened beverages was associated with a higher risk of obesity [18]. However, given the differences in food culture and habits, DPs identified in non-Chinese populations may not represent the dietary intake characteristics in the Chinese population. Moreover, China is undergoing a major nutrition transition owing largely to the rapid economic development over the past four decades [19]. Consumption of a traditional Chinese diet that emphasizes cereal grains and legumes has dropped, whereas edible oil, meat, and sugar-sweetened beverage intakes associated with a Western-style diet have increased in the Chinese population in China [20,21,22,23]. To inform nutrition policies, it is crucial to identify DPs that are consistently associated with a reduced risk of obesity in the Chinese population. From there, interventions may be developed and implemented to facilitate the application of healthful DPs to reduce the risk of obesity in the Chinese population.

To the best of our knowledge, no meta-analysis has ever been conducted on this topic in the Chinese population. We have previously reviewed the relationship between a priori dietary scores and weight outcomes in the Chinese population and found inconsistent results, partly attributed to the unique characteristics of the diet consumed by Chinese cohorts with geographical, cultural, and socioeconomic differences [24]. Therefore, the a posteriori approach that summarizes major dietary components based on dietary data from the cohorts may provide a better characterization of the diet consumed by the Chinese population. In this study, we will focus on studies that report a posteriori DPs and their associations with obesity and weight management.

## 2. Materials and Methods

### 2.1. Inclusion Criteria

This is a systematic review and meta-analysis related to DPs and obesity outcomes in the Chinese population. Cross-sectional studies, longitudinal cohort studies, case-control studies, and randomized controlled trials (RCTs) were included, while reviews, meta-analyses, and editorials were excluded. Studies were included if they enrolled Chinese adults aged over 18 years residing either inside or outside China. Studies were excluded if they only included children, pregnant or lactating women, or non-Chinese populations. These groups were excluded because they have different dietary and nutrition requirements and thus are more appropriate to be reviewed separately. The exposure of interest was any DP that described the characteristics of the overall diet and was formed by a posteriori factor analysis. Therefore, studies based on a priori DP-derived dietary indexes, such as the MDS, were excluded. We have previously reviewed how these a priori dietary indexes were related to weight status in Chinese adults [24]. Studies that targeted the intake of specific nutrients or foods without characterizing the overall DP were also excluded. The primary outcome of interest was overweight and obesity as assessed by body mass index (BMI). The BMI cut-offs were <18.5 kg/m^2^ for underweight, 18.5–23.9 kg/m^2^ for normal weight, 24–27.9 kg/m^2^ for overweight, and ≥28 kg/m^2^ for obesity for Chinese cohorts, unless specified otherwise in individual studies. These cut-offs were established by the Working Group on Obesity in China because obesity-related metabolic risk occurs at a lower BMI in the Chinese population [25,26]. Secondary outcomes of interest were measurements of central obesity such as waist circumference (WC), waist-to-hip ratio, as well as body weight and weight change over time. Studies were included if they contained clear statistical analyses of the relationship between the exposures and outcomes of interest with the odds ratio (OR), prevalence ratio (PR), or risk ratio (RR) and/or *p*-value presented and confounding factors adequately addressed in the statistical models.

### 2.2. Search Strategies

We selected PubMed and CINAHL Complete (EBSCO) for our primary databases to conduct comprehensive searches for research articles published between 1 January 2000 and 1 February 2022. The terms used in the search strategies included MeSH terms and keywords. “China” and “Chinese” were used for keywords and MeSH to define the population; for the DPs, keywords including “dietary pattern” as well as MeSH terms and CINAHL subject headings including “diet”, “feeding behavior”, and “food habits” were used; for outcomes, terms including “body mass index”, “overweight”, “obese”, “waist circumference”, and “weight gain” were used. Truncation was also used as necessary, such as (diet* AND pattern*), due to a lack of controlled vocabulary for this concept. For PubMed, searches used a combination of MeSH and keywords. The title/abstract [tiab] tag was used with keywords. CINAHL searches were combinations of CINAHL subject headings and keywords, and no field codes were used to allow for a wider breadth of search results. In addition, using Scopus for citation tracing purposes, searches were performed on studies that met inclusion criteria to further ensure research comprehensiveness and locate studies that might not have been retrieved using the predefined search algorithms. Search results were imported into Endnote (Clarivate Analytics, London, UK) for study selection.

### 2.3. Study Selection

We first removed duplicates identified across databases using Endnote (Clarivate Analytics, London, UK). Thereafter, two researchers (Karen Jiang and Xinyin Jiang) screened the articles based on titles and abstracts and excluded those found to be irrelevant. These researchers further conducted a full-text review of the remaining papers based on the inclusion and exclusion criteria. Disagreements between the two researchers were resolved following a discussion to reach a consensus, or, if consensus was not reached, a senior researcher (Liang Wang) was invited to arbitrate.

### 2.4. Statistical Analysis

The meta-analysis combined the multivariable OR, PR, and RR of DPs comparing the highest versus lowest quantile based on a random-effects model [27]. When a multivariable adjusted OR/PR/RR was unavailable for the specific outcome, an unadjusted OR was calculated. Weighting of each study was based on the standard error for the OR/PR/RR using an inverse variance method [27]. Studies were grouped according to different DPs and different weight outcomes (e.g., BMI, waist circumference, visceral obesity, waist-to-hip ratio). Heterogeneity was assessed with I^2^ statistics [28]. Publication bias was assessed using the funnel plot and Egger’s linear regression test [29]. Sensitivity tests were conducted by removing studies that have a differential characterization of a DP and studies that only have an unadjusted OR for a specific outcome. Subgroup analyses were conducted for longitudinal and cross-sectional studies, respectively. Analyses were conducted using Review Manager 5.4 by the Cochrane Collaboration and the Metafor package for R Statistical Software (v4.1.2; R Core Team 2021, the R foundation, Indianapolis, IN, USA) [30].

### 2.5. Study Quality Assessment

We assessed the quality of studies using the National Institutes of Health Quality Assessment Tool for Observational Cohort and Cross-Sectional Studies since there were no RCTs identified from our search [31]. The assessment tool includes 14 equally weighted criteria; thus, the highest possible score indicating a high-quality study is 14. Scores less than 7 indicated a high risk of bias, 7–10 showed a moderate risk, and 11–14 was considered as low risk.

## 3. Results

**Figure 1:**
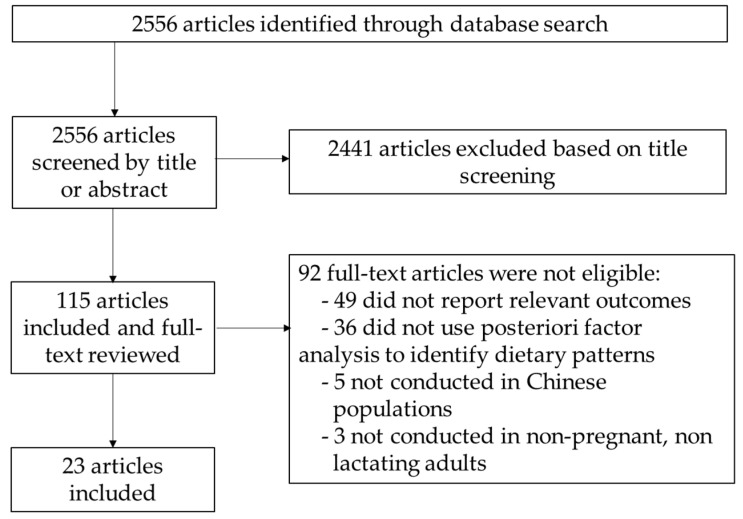
Flowchart for the article selection process.

We identified 2556 articles from the initial database search (Figure 1). After screening based on title or abstract, 115 papers remained and were included in the full-text review. Of those, 92 were found to have irrelevant outcomes, topics, or study populations and were therefore excluded from the review. After the search and selection process, 23 articles met all inclusion criteria and were included in the review (Table 1 and more details in Supplementary Table S1). Of these 23 articles, 18 were cross-sectional studies [32,33,34,35,36,37,38,39,40,41,42,43,44,45,46,47,48,49], and 5 were prospective cohort studies [50,51,52,53,54]. Some primary DPs identified included the traditional Chinese DP (16 studies) [35,37,39,40,41,42,43,44,47,48,49,50,51,52,53,54], modern/Western DP (12 studies) [33,34,36,37,40,42,44,48,50,51,53,54], meat/animal protein DP (14 studies) [33,34,35,37,38,39,40,43,45,46,47,49,52,54], and plant food/vegetarian DP (10 studies) [33,34,35,36,38,39,45,46,49,52]. Overall, 9 studies included BMI [33,35,40,44,47,48,49,50,53] and 8 included WC [33,34,35,40,44,47,48,53] as a multivariate-adjusted continuous outcome, 15 assessed the risk of being underweight or overweight and obese [34,35,36,37,38,39,41,42,44,47,48,49,50,51,54], and 6 examined the risk of central or abdominal obesity [32,40,43,44,46,51]. Two longitudinal studies investigated weight gain as an outcome [45,52]. Regarding participants, 2 studies had fewer than 1000 [32,45], 19 studies had 1000–10,000 [33,34,35,36,37,39,40,41,42,43,46,47,48,49,50,51,52,53,54], and 2 studies had over 10,000 [38,44]. Twenty studies were conducted in mainland China [34,35,36,37,39,40,41,42,43,44,45,46,47,48,49,50,51,52,53,54], two were conducted in the Taiwan region [32,38], and one was conducted in the Hong Kong special administrative region (SAR) of China [33]. Eight studies used 24 h dietary recall [42,47,48,49,50,51,53,54] and fifteen used a food frequency questionnaire (FFQ) [32,33,34,35,36,37,38,39,40,41,43,44,45,46,52] to collect dietary information.

**Figure 2:**
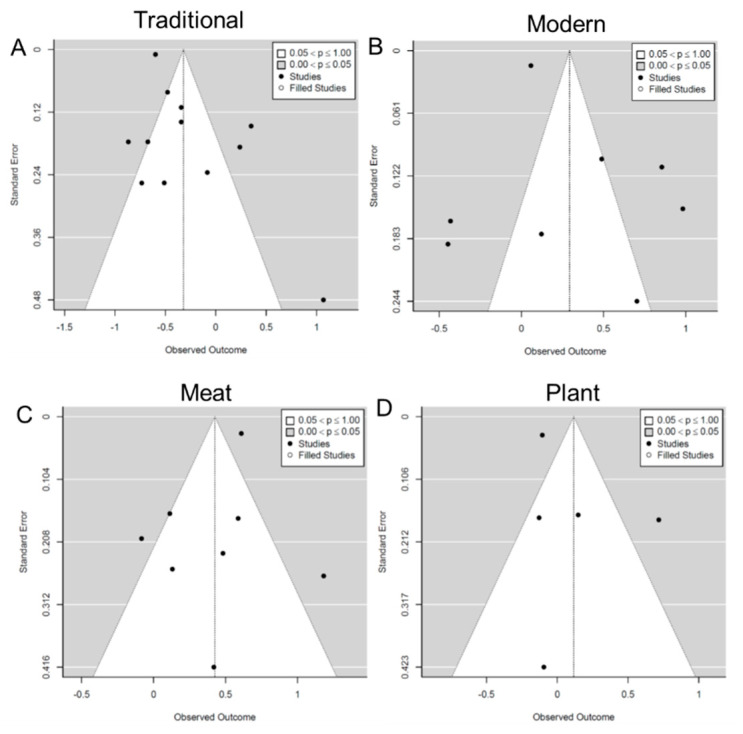
Funnel plot of pooled odds ratios (ORs) with 95% confidence interval (CI) for the highest versus lowest quantile of the different dietary patterns for overweight/obesity. (**A**) traditional dietary pattern; (**B**) modern dietary pattern; (**C**) meat/animal protein dietary pattern; (**D**) plant food/vegetarian dietary pattern.

Five included studies (22%) were considered to have a low risk of bias, all of which were longitudinal studies. In comparison, the other 18 studies (78%) were determined as having a moderate risk of bias, and all of them were cross-sectional studies (Supplementary Table S2). The risk of bias in the cross-sectional studies was mostly related to the lack of temporal separation between DPs and obesity outcomes. The funnel plots (Figure 2) and Egger’s tests did not identify significant asymmetry (*p* = 0.07–0.98), suggesting that there was no significant publication bias.

### 3.1. The “Traditional Chinese” DP and Weight Status

Out of 23 studies, 16 identified the “traditional Chinese diet” as a DP; 5 of them were longitudinal [50,51,52,53,54] while the remaining 11 were cross-sectional studies [35,37,39,40,41,42,43,44,47,48,49]. Although there are some variations of how this DP is defined in different studies, in general, this pattern includes starchy food such as rice, wheat, and tubers, as well as vegetables and high-protein foods, especially pork. Three longitudinal studies were not included in the meta-analysis because they treated the traditional DP as a continuous variable [51] or did not have overweight/obesity as an outcome [52,53]. One cross-sectional study by Ye et al. [43] was not included in the meta-analysis because it did not have overweight/obesity as an outcome.

**Figure 3:**
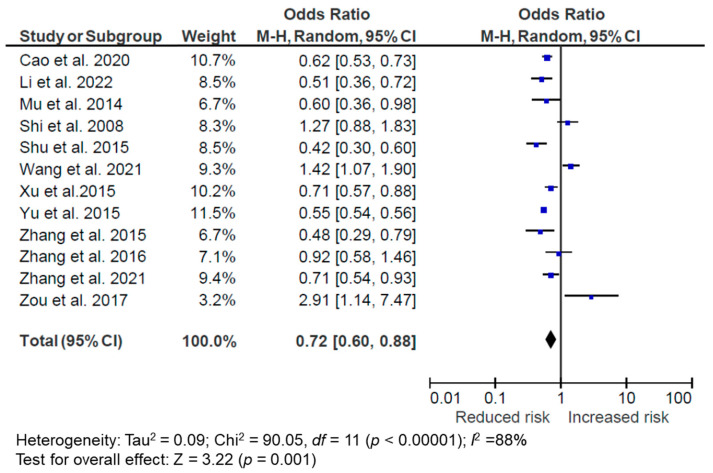
Forest plot of pooled odds ratios (ORs) with 95% confidence interval (CI) for the highest versus lowest quantile of the traditional dietary pattern for overweight/obesity [35,37,39,40,41,42,44,47,48,49,50,54].

For the 12 included studies (2 longitudinal and 10 cross-sectional), 8 studies showed a negative association, 2 showed no association, and 2 showed a positive association between the traditional DP and overweight/obesity. All of them were conducted in mainland China. The meta-analysis suggests that overall, following the traditional DP is associated with a reduced risk of overweight/obesity (OR = 0.72, 95% CI: 0.60, 0.88, *p* = 0.001, *I*^2^ = 88%, *n* = 12) (Figure 3). In the subgroup analyses, cross-sectional studies (OR = 0.75, 95% CI: 0.58, 0.98, *p* = 0.04, *I*^2^ = 90%, *n* = 10) and longitudinal studies (OR = 0.64, 95% CI: 0.56, 0.74, *p* < 0.001, *I*^2^ = 0%, *n* = 2) both demonstrated an inverse association between the traditional DP and reduced risk of overweight/obesity. There were significant heterogeneities among studies, particularly among the cross-sectional studies. We conducted sensitivity tests by excluding studies that could only provide a raw or unadjusted OR [40,42], but the association was not materially changed (OR = 0.77, 95% CI: 0.61, 0.98, *p* = 0.03, *I*^2^ = 89%, *n* = 10). When excluding two studies that had some variations in the characterization of the traditional DP, the association was also not attenuated (OR = 0.74, 95% CI: 0.58, 0.86, *p* = 0.02, *I*^2^ = 85%, *n* = 10). In one of these studies, Zhang et al. [48] defined the traditional DP as containing wheat, cake, and oil in the 2010–2012 National Nutrition Survey in the province of Yunnan, and did not find an association between this DP and overweight/obesity. The other study by Yu et al. [44] subdivided the traditional DP into traditional southern and traditional northern DPs. However, neither contained similar components of traditional DP with staple food, vegetables, and high-protein foods compared to other studies. This study found that the traditional northern DP with wheat, high-protein foods, and fresh fruits was associated with higher risks of general (PR = 1.05, 95% CI: 1.02, 1.09) and central obesity (PR = 1.17, 95% CI: 1.15, 1.18) compared to the traditional southern DP with high rice and low wheat intake [44].

For the longitudinal studies that were not included in the meta-analysis, maintaining a traditional DP over time was related to a lower risk of obesity (*β* = −0.10, 95% CI: 0·01, 0·07) [55], greater BMI decrease (*β* = −0.23, 95% CI: −0.44, −0.02) [53] or less weight gain (*β* = −2.18, 95% CI: −2.91, −1.45) [52]. The cross-sectional study by Ye et al. [43] that was not included in the meta-analysis demonstrated that a healthy traditional DP was associated with a lower risk of abdominal obesity (OR = 0·52, 95% CI: 0.41, 0.67).

### 3.2. The “Modern” DP and Weight Status

Out of 23 studies, 12 identified “modern” or “Western” as a DP. Since key components in these two identified patterns are similar, including fast food, milk, processed meat, and deep-fried food, they were clustered together as the same DP in the review and meta-analysis. Four were longitudinal [50,51,53,54] while the remaining eight were cross-sectional studies [33,34,36,37,40,42,44,48]. The longitudinal study by Xu et al. [53] and the cross-sectional study by Chan et al. [33] were not included in the meta-analysis due to the lack of an overweight/obesity outcome. The longitudinal study by Li et al. [51] and the cross-sectional study by Chen et al. [34] were also excluded because the DP was scored as a continuous variable.

**Figure 4:**
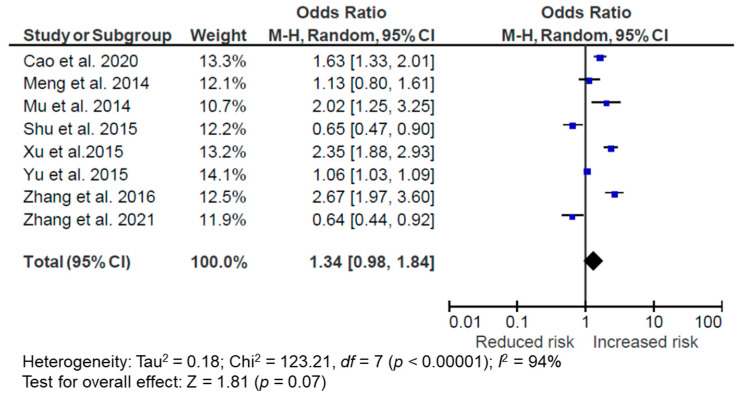
Forest plot of pooled odds ratios (ORs) with 95% confidence interval (CI) for the highest versus lowest quantile of the modern/Western dietary pattern for overweight/obesity [36,37,40,42,44,48,50,54].

Overall, 8 studies were included in the meta-analysis (2 longitudinal and 6 cross-sectional), with 5 of them demonstrating a positive association, 2 demonstrating a negative association, and 1 demonstrating no association between the modern DP and overweight/obesity. All of them were conducted in mainland China. Overall, following a modern DP was associated with a higher OR of overweight/obesity but did not reach statistical significance (OR = 1.34, 95% CI: 0.98, 1.84, *p* = 0.07, *I*^2^ = 94%, *n* = 8) (Figure 4). Subgroup analyses suggested a similar OR in cross-sectional studies (OR = 1.46, 95% CI: 0.97, 2.19, *p* = 0.07, *I*^2^ = 95%, *n* = 6) but a null association in the longitudinal studies (OR = 1.03, 95% CI: 0.41, 2.60, *p* = 0.94, *I*^2^ = 95%, *n* = 2). However, it should be noted that there were only two longitudinal studies included in this analysis, and the longitudinal study by Zhang et al. [35] characterized the modern DP with fruits, dairy, and processed food, where the fruit intake might have attributed to the inverse relationship between the modern DP and overweight/obesity in that study (overweight: OR = 0.76, 95% CI: 0.63, 0.91; obesity: OR = 0.64, 95% CI: 0.44, 0.90). In sensitivity analyses, the relationship between the modern DP and overweight/obesity was not modified by removing studies that could only provide an unadjusted OR [40,42] in the meta-analysis (OR = 1.36, 95% CI: 0.98, 1.91, *p* = 0.07, *I*^2^ = 92%, *n* = 6). Removing the study by Yu et al. [44], which had a differently defined modern/Western DP with fresh fruits and protein products, did not change the OR magnitude much but further reduced the statistical significance of the association (OR = 1.39, 95% CI: 0.92, 2.10, *p* = 0.12, *I*^2^ = 93%, *n* = 7). The I^2^ statistics suggest that these studies were heterogeneous.

For the two longitudinal studies that were not included in the meta-analysis, maintained or increased adoption of the modern DP over time was related to a higher risk of obesity [32] and greater BMI, weight, and WC increases [34]. The cross-sectional studies by Chan et al. and Chen et al. [34,46] excluded from the meta-analysis did not find statistically significant associations between the modern DP and BMI or sarcopenic obesity. However, Chan et al. [46] suggested that the modern DP (named as the “snacks–drinks–milk” DP in the study) was associated with a lower waist-to-hip ratio (*β* = −0.004, 95% CI: −0.007, −0.001) in older adults aged over 65 living in Hong Kong.

Overall, three out of four longitudinal studies indicated that maintained or increased adoption of the modern DP over time was related to higher BMI or obesity outcomes, except for the longitudinal study by Zhang et al. [54], which had a different definition of the modern DP illustrated above. Findings from the eight cross-sectional studies were not consistent, with five of them showing null associations and three showing a positive association between the modern DP and overweight/obesity or BMI.

### 3.3. The Meat/Animal Protein DP and Weight Status

Out of 23 studies, 14 identified “meat/animal protein” as a DP with meat and other animal proteins as its main components [33,34,35,37,38,39,40,43,45,46,47,49,52,54]. However, there were several variations in the factors included in the DP, with some studies also including plant proteins [43,47], alcoholic beverages [39,52], processed food [35,38,46], or starch [40,45] in the DP. Two of the studies were longitudinal, while the remaining twelve studies were cross-sectional.

**Figure 5:**
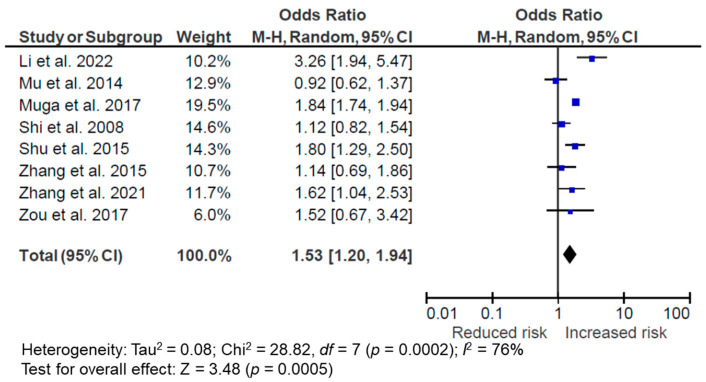
Forest plot of pooled odds ratios (ORs) with 95% confidence interval (CI) for the highest versus lowest quantile of the meat/animal protein dietary pattern for overweight/obesity [35,37,38,39,40,47,49,54].

One longitudinal study by Shi et al. [52] and five cross-sectional studies by Zhang et al. [45], Zhang et al. [46], Chan et al. [33], Chen et al. [34], and Ye et al. [43] were not included in the meta-analysis due to the lack of overweight/obesity outcomes or the DP being scored as a continuous variable. The meta-analysis suggested that a meat/animal protein DP was associated with an increased risk of being overweight/obese (OR = 1.53, 95% CI: 1.20, 1.94, *p* = 0.0005, *I*^2^ = 76%, *n* = 8) (Figure 5). The subgroup analysis with only cross-sectional studies did not affect the association (OR = 1.52, 95% CI: 1.16, 1.99, *p* = 0.002, *I*^2^ = 79%, *n* = 7). However, it should be noted that three of the included studies also incorporated starch [40] or processed food [35,38] in the meat/animal protein DP. When these studies were removed from the sensitivity test, the association between the DP and overweight/obesity disappeared (OR = 1.17, 95% CI: 0.96, 1.42, *p* = 0.11, *I*^2^ = 0%, *n* = 5), suggesting that the positive association was partly driven by these studies with non-meat/non-high-protein components in the DP. There were substantial heterogeneities among the studies.

Within all of the 14 studies that included the meat/animal protein DP and any weight outcomes, 5 studies only included animal proteins in the DP [33,34,37,49,54]. One cross-sectional study by Chan et al. [33] found the DP to be associated with a higher BMI, and one longitudinal study by Zhang et al. [54] showed that a high initial score and a slight decrease trajectory of the meat DP was associated with a higher risk of overweight/obesity (OR = 1.63; 95% CI: 1.04, 2.54). Three other cross-sectional studies [34,37,49] did not find an association between this DP and weight status. Two cross-sectional studies [43,47] included both animal and plant proteins in the DP and neither of them found a statistically significant association between the DP and overweight or obesity. Shi et al. [39,52] conducted a cross-sectional and later a longitudinal study on the Jiangsu Nutrition study cohort and did not find an association between a “macho” or meat and alcohol DP and weight status or weight gain over time. Three cross-sectional studies included both animal proteins and processed food in the DP, and all of them found a positive association with a higher risk of being obese [35,38,46]. Lastly, two cross-sectional studies also included starch in the DP, with one of them demonstrating increased risks for higher BMI and abdominal obesity (OR = 1.67, 95% CI: 1.19, 2.34) in middle-aged adults and the other one showing less weight gain after marriage in newly wed couples (*β* = −1.21; 95% CI: −2.32, −0.11; *p* = 0.03) [40,45].

### 3.4. The Plant Food/Vegetarian DP and Weight Status

**Figure 6:**
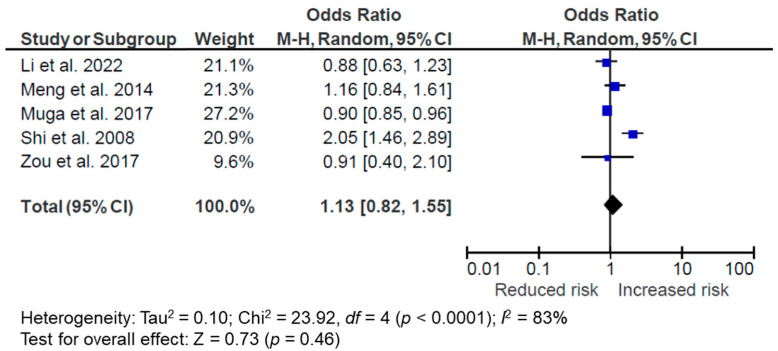
Forest plot of pooled odds ratios (ORs) with 95% confidence interval (CI) for the highest versus lowest quantile of the meat/animal protein dietary pattern for overweight/obesity [35,36,38,39,49].

Out of 23 studies, 10 identified a plant food or vegetarian DP [33,34,35,36,38,39,45,46,49,52]. The main components are fruits and vegetables; some also include other plant foods such as starchy vegetables and grains, and egg and dairy as in the ovo-lacto vegetarian DP. Some studies also include animal products in the DP. Only one of them was longitudinal [52], while the rest were cross-sectional [33,34,35,36,38,39,45,46,49]. Five studies (all cross-sectional) were included in the meta-analysis [35,36,38,39,49], while the other five were excluded due to no overweight/obesity outcome or the DP being treated as a continuous variable [33,34,45,46,52]. The meta-analysis suggests no association between this DP and overweight/obesity (OR = 1.13, 95% CI: 0.82, 1.55, *p* = 0.46, *I*^2^ = 83%, *n* = 5) (Figure 6). The sensitivity test that excluded studies with non-plant components in the DP [35,39] did not modify the result (OR = 0.93, 95% CI: 0.83, 1.04, *p* = 0.32, *I*^2^ = 11%, *n* = 3).

Within the 10 studies that investigated the plant food/vegetarian DP and any weight or obesity outcomes, for the 6 studies that only had plant source foods in the DP, Muga et al. [38] included only fruits and vegetables in the DP and identified it to be associated with a lower risk of being overweight (OR = 0.91, 95% CI: 0.85, 0.97) or obese (OR = 0.85, 95% CI: 0.78, 0.92). Meng et al. [36] included both staple foods and vegetables in the DP and identified a higher risk of obesity (OR = 2.67). The others did not find an association between the DP and obesity [33,45,46,49]. Two cross-sectional studies identified an ovo-lacto vegetarian DP. One of the studies identified a negative relationship between this DP and sarcopenic obesity (OR = 0.79, 95% CI: 0.65, 0.97) [34], while the other did not find a significant association between the DP and overweight/obesity [35]. In the cross-sectional and follow-up longitudinal studies by Shi et al. [33,37], the vegetable-rich DP was also characterized by milk, egg, and fish, and the studies identified this DP to be associated with a higher risk of general obesity (PR = 2.06, 95% CI: 1.46, 2.89) and weight gain (*β* = 1.00, 95% CI 0.25, 1.74).

### 3.5. Other DP and Weight Status

There were also DPs that seem to be unique to the specific cohort and thus could not be compared across studies. For example, Cempaka et al. [32] identified a dysregulated iron metabolism-related pattern and found it to be associated with central obesity (OR = 1.57, 95% CI: 1.05, 2.34). A few studies identified snacks and beverages in the DP and found them to be associated with a lower waist-to-hip ratio in men (*β* = −0.004, 95% CI: −0.007, −0.001) [34], higher risk of obesity (OR = 3.26, 95% CI: 1.37, 7.69) [49], or no association with weight status or obesity [43,47]. Sweets were identified in a few studies as a DP, with either an association with more weight gain after marriage (β = 2.94, 95% CI: 0.75, 5.15) in men in one study [45] or no association in two others [33,37].

## 4. Discussion

In the current systematic review and meta-analysis, we found that the traditional Chinese DP was associated with a lower risk of overweight/obesity while other DPs, including modern/Western, meat/animal protein, and vegetarian DP, had inconsistent results with weight and obesity outcomes in the Chinese population.

The traditional Chinese DP demonstrates consistent benefits in lowering the risk of obesity across different studies in both cross-sectional and longitudinal settings. This DP is composed of grains (especially rice), vegetables, and high-protein foods (especially pork). This a posteriori DP has a structure that is consistent with the recommendation of a priori dietary guidelines, such as the Chinese Food Pagoda (CFP), which recommends the inclusion of whole grains and beans, tubers, non-starchy vegetables (especially dark vegetables), fruits, dairy, soybeans, high-quality protein, and nuts and seeds, while limiting red meat, cooking oil, sodium, added sugar, and alcohol intake [56]. It should be noted that there are substantial differences in dietary habits across different regions of China. The “grain, vegetable, and meat”-based traditional Chinese diet seems to better resemble the diet of the southern part of China. Some researchers have advocated for the benefit of the so-called “Jiangnan” diet, meaning the Southern River diet, typically consumed around the downstream reaches of the Yangtze River [55]. This diet includes high consumption of vegetables and fruits in season, freshwater fish and shrimp, and legumes; moderate consumption of whole-grain rice, plant oil, and red meat; and low consumption of salt or millet wine. This style also prefers steaming or boiling and lukewarm-fire frying for cooking [55]. This style shares some similarities with the Mediterranean style diet, emphasizing vegetables, fruits, whole grains, healthy plant oil, and moderate consumption of red meat. The Mediterranean diet has been consistently shown in different studies to benefit weight management and cardiovascular disease prevention [57,58]. In contrast, the diet in northern China is traditionally full of starchy vegetables and wheat products, and was associated with an increased risk of obesity in one of the studies [47].

There have been tremendous changes in eating habits in China since the opening up of the country in the late 1970s. Some notable characteristics of Westernization are the dramatic increases in protein (meat) consumption, snacking behavior, fast food, processed food (i.e., foods that require multiple processes in production and contain multiple added ingredients), and sugar-sweetened beverage (SSB) consumption [59,60,61]. However, these changes have not been consistently demonstrated to impact body weight in different studies. The “modern” or “Western” DP demonstrated a higher OR in association with overweight/obesity but did not reach statistical significance. The different study designs may have partly contributed to the observed difference in the association. While three out of four longitudinal studies demonstrated the risk of this DP for increased weight gain and obesity-related issues in the long term [50,51,53], cross-sectional studies that captured a snapshot of intake of this DP had varied associations with obesity in general [33,34,36,37,40,42,44,48]. This suggests that the negative impact of the modern DP may become more apparent through cumulative exposure. Conversely, breaking the habit of following the modern DP and improving dietary quality may offer long-term benefits in weight outcomes, as was demonstrated in the study of both the Nurses’ Health Study (NHS) and the Health Professionals Follow-Up Study (HPFS) cohorts in the U.S. [62]. The inconsistent findings from cross-sectional versus longitudinal studies also highlight the importance of careful interpretation of cross-sectional results, with the understanding of the limitation of dietary data collection at one time-point when there may be dynamic changes in dietary habits over time, such as during the drastic nutrition transition in China.

There are substantial increases in protein (especially animal protein consumption) in China, with per capita meat consumption increasing by 74% in urban and 134% in rural China from 1981 to 2012 during the rapid economic growth of the country [63]. Animal proteins are unsurprisingly frequent in a posteriori DPs identified in the included studies in the current review. However, how they were clustered or loaded together with other dietary components in a DP was different across studies, and this heterogeneity may have contributed to inconsistent results among studies. In general, it seems that consumption of animal protein itself is not related to a higher BMI or obesity risk in most studies, and the null relationship also applies to a high protein diet with both animal and plant proteins. However, people with high meat consumption often have high processed food consumption as well, as demonstrated in three cross-sectional studies, and all of them suggest a positive relationship between this high-meat, high-processed food DP and obesity [35,38,46]. Food from an animal source provides high-quality protein and essential nutrients such as vitamin B_12_ and iron. Its intake may help prevent protein deficiency and facilitate the recovery of those with acute injury or diseases. The estimated population affected by iron deficiency anemia in 2008 was 208 million in China [64]. Meat provides heme iron, a more bioavailable form of iron that may help lessen the disease burden of iron deficiency. An emphasis on portion size control following the CFP may be warranted to maximize the benefit of meat while minimizing the potential risk of meat consumption with calorie excess and metabolic syndrome. Although not often distinguished in DP identification, whether animal protein from different sources may have differential relationships with weight outcomes requires further investigation. In non-Chinese populations, an observational study suggested that total meat intake was not associated with weight change over time in older adults, yet the effects of subtypes of the meat were different and sex specific [65]. However, an RCT found that consumption of pork, chicken, or beef did not yield different changes in adiposity [66].

A plant-based diet has been increasingly proposed for chronic disease prevention. Plant-based foods such as fruits and vegetables, whole grains, nuts and seeds, and legumes provide the human body with essential vitamins and minerals, as well as antioxidant phytochemicals and dietary fibers. However, the relationship between a vegetarian diet and weight management has not been evident in this systematic review and meta-analysis. This may again be partly attributed to the heterogeneity in the DP as to what plant foods are included and whether they are major calorie providers in the diet. Such heterogeneity is consistent with recent studies on the quality of different plant foods. While a healthful plant-based diet with fruits and vegetables, whole grains, legumes, nuts and seeds, plant oil, coffee, and tea is related to a reduced risk of coronary heart disease, an unhealthful plant-based diet full of fruit juices, refined grains, French fries, and desserts is related to an increased risk in the NHS and HPFS cohorts [67]. These results underscore that a high-quality plant-based diet should be encouraged.

There are limitations of this study. First, the heterogeneity of DPs in different studies have led to inconsistent results and posed a challenge to the meta-analysis. Generalization of the results to different populations and contexts requires particular caution. Most of the studies were cross sectional. Therefore, it is unclear whether a DP would have a positive influence on weight change over time, though limited longitudinal studies suggest this may be the case. Nevertheless, this is the first systematic review and meta-analysis on DPs and weight outcomes focused on the Chinese population. All but three studies included in the systematic review were conducted within mainland China and thus represent the relationship between DP and obesity during a drastic societal nutrition change in China. Conversely, this systematic review and meta-analysis may not represent the DP and weight relationship in Chinese populations residing outside East Asia. The consistent beneficial effect of the traditional Chinese DP on weight management pinpoints a direction for public health promotion to address the rapidly increasing prevalence of obesity in China.

## 5. Conclusions

In conclusion, a traditional Chinese DP characterized by rice or other grains, vegetables, and meat or plant protein is related to a lower risk of obesity in the Chinese population in China. Substituting this DP for the modern DP characterized by fast food, deep-fried food, and processed meat may be a useful public health intervention to address the obesity epidemic in China.

## Figures and Tables

**Table 1:** Summary of characteristics of included studies on dietary patterns and obesity or weight outcomes.

Number	Pattern	Study Type	Participants	Population	Outcome	Food Measurements	Associations Identified
Cao et al., 2020 [50]	Trajectory of the “traditional” DP and “modern” DP	Longitudinal	6943 (48.4% males)	Adults aged over 20 years in the China Health and Nutrition Survey (CHNS) between 1991 and 2009	BMI, overweight/obesity in 2009	3 day 24 h recall	High and stable traditional DP trajectory: ↓ BMI; high and rapid increase in the modern DP: ↑ BMI
Cempaka et al., 2019 [32]	“Dysregulated iron metabolism-related” DP	Cross sectional	208 (50.4% males)	Taiwanese adults aged 20–65 years	Central obesity, fat mass	FFQ	Central obesity; visceral fat mass (%)
Chan et al., 2012 [33]	“Vegetables–fruit” DP; “snacks–drinks–milk products” DP; “meat–fish” DP	Cross sectional	3707 (52.5% males)	Adults aged 65 years and above living in Hong Kong	BMI, WC, HC, waist-to-hip ratio	FFQ	Meat–fish DP: ↑ BMI, waist-to-hip ratio, and WC in men; ↑ BMI, WC, and HC in women; “snacks–drinks–milk products” DP: ↓ waist-to-hip ratio in men
Chen et al., 2021 [34]	“Lacto-ovo-vegetarian” DP; “meat-fish” DP; “junk food” DP	Cross sectional	3795 (37.2% males)	Community-dwelling older adults aged over 60 years in Shenyang, Liaoning province	Sarcopenic obesity, WC, obesity	FFQ	Lacto-ovo-vegetarian DP: ↓ sarcopenic obesity
Li et al., 2017 [51]	Mean cumulative DP scores during 1991–2011 for “traditional” and “modern” DPs	Longitudinal	9499 (48% males)	CHNS between 1991 and 2011	Overweight/obesity (BMI > 25 kg/m^2^), abdominal obesity in 2009	3 day 24 h recall	Traditional DP: ↓ general and abdominal obesity; modern DP: ↑ general and abdominal obesity
Li et al., 2022 [35]	“Animal-based and processed food” DP; “traditional food” DP; “ovo-lacto vegetarian food” DP	Cross sectional	1136 (100% males)	Males aged over 65 years in Sichuan province	BMI, overweight/obesity, WC	FFQ	Traditional DP: ↓ overweight/obesity; animal-based and processed food DP: ↑ overweight/obesity
Meng et al., 2014 [36]	“Western food” DP; “high-protein and -calcium” DP; “fruits and snacks” DP; “staple food and vegetables” DP	Cross sectional	1535 (47.4% males)	Adults aged ≥ 18 years old in Shanghai	Overweight/obesity	FFQ	Staple food and vegetables DP: ↑ obesity
Mu et al., 2014 [37]	“Western food” DP; “high-protein and -calcium” DP; “calcium food” DP; “Chinese traditional” DP	Cross sectional	1319 (38.7% males)	College freshmen aged 16–20 years in Anhui province	Overweight/obesity	FFQ	Western food DP: ↑ overweight/obesity; traditional DP: ↓ overweight/obesity
Muga et al., 2017 [38]	“Vegetable–fruit” DP, “processed meat” DP	Cross sectional	62,965 (52% males)	Taiwanese adults aged over 40 years	Overweight/obesity	FFQ	Vegetable–fruit DP: ↓ overweight/obesity; meat and processed DP: ↑ overweight/obesity
Shi et al., 2008 [39]	“Traditional” DP; “vegetable-rich” DP; “macho” DP; “sweet tooth” DP	Cross sectional	2849 (45.9% males)	Adults aged over 20 years in the Jiangsu Nutrition Study (JIN)	Overweight/obesity	FFQ	Vegetable-rich DP: ↑ general obesity
Shi et al., 2011 [52]	“Traditional” DP; “vegetable-rich” DP; “macho” DP; “sweet tooth” DP	Longitudinal	1231 (41.4% males)	JIN 2002–2007	Weight gain during the survey period	FFQ	Traditional DP: ↓ weight gain; vegetable-rich DP: ↑ weight gain
Shu et al., 2015 [40]	“Animal food” DP; “traditional Chinese” DP; “Western fast-food” DP; “high-salt” DP	Cross sectional	2560 (53% males)	Adults aged 45–60 years from Zhejiang province	BMI, WC, waist-to-hip ratio (WHR), abdominal obesity	FFQ	Animal DP: ↑ BMI, WC, and abdominal obesity; traditional Chinese DP: ↓ BMI, WC, and abdominal obesity;
Wang et al., 2021 [41]	“Traditional” DP; “fruit–egg” DP; “nut–wine” DP	Cross sectional	1739 (46.2% males)	Adult participants aged over 18 years in Jiangsu province	Overweight/obesity	FFQ	Traditional DP: ↑ overweight and obesity in men but not in women
Xu et al., 2015 [42]	“Traditional” DP; “modern” DP	Cross sectional	2745 (47.4% males)	2009 CHNS participants aged ≥ 60 years	Obesity	3 d food recalls	Traditional DP: ↓ overweight and general obesity; modern DP: ↑ central obesity in men, ↓ underweight in women
Xu et al., 2016 [53]	“Traditional” and “modern” DPs as above over four survey years	Longitudinal	6348 (47.3% males)	CHNS 2004–2011 waves of participants aged ≥ 60 years	BMI, weight and WC changes over four survey years	3 d food recalls	Traditional DP: ↓ BMI, weight, and WC; modern DP: ↑ BMI, weight, and WC
Ye et al., 2018 [43]	“Healthy traditional” DP; “animal and plant protein” DP; “condiments” DP; “fruits, eggs, and juice” DP; “alcohol, milk, and tea” DP	Cross sectional	3376 (41.4% males)	Adult participants aged over 35 years in Nanjing	Abdominal obesity	FFQ	Healthy traditional DP: ↓ abdominal obesity
Yu et al., 2015 [44]	“Traditional southern” DP; “traditional northern” DP; “Western” DP	Cross sectional	474,192 (59% males)	Adults aged 30–79 years from the China Kadoorie Biobank	BMI, WC, general obesity, central obesity	FFQ	Traditional southern DP: ↓ general and central obesity; Traditional northern DP: ↑ general obesity and central obesity; Western DP: ↑ general obesity and central obesity;
Zhang et al., 2012 [45]	“Vegetable” DP; “sweets and fats” DP; “legume” DP; “poultry, beef, and mutton” DP	Cross sectional	556 (50.5% males)	Newlywed couples aged under 35 years in Shanghai	Weight gain	FFQ	Sweets and fats DP: ↑ weight gain after marriage in men; poultry, beef, and mutton DP: ↓ weight gain after marriage
Zhang et al., 2014 [46]	“Animal food” DP; “plant food” DP; “seafood” DP	Cross sectional	2116 (46.6% males)	Adults aged over 18 years in the “China National Nutrition and Health Status Monitoring” cohort	Abdominal obesity	FFQ	Animal food DP: ↑ abdominal obesity; seafood DP: ↓ abdominal obesity
Zhang et al., 2015 [47]	“Traditional southern” DP; “traditional northern” DP; “snack” DP; “high-protein” DP	Cross sectional	2363 (100% females)	Women aged 18–44 years in the 2011 CHNS	Obesity, BMI, WC	3 d food recalls	Traditional southern DP: ↓ general and abdominal obesity; traditional northern DP: ↑ general and abdominal obesity
Zhang et al., 2016 [48]	“Modern” DP; “traditional” DP; “tuber” DP	Cross sectional	1604 (41.4% males)	Adults aged 18–80 years in Yunnan province	Obesity, BMI, WC	3 d food recalls	Modern DP: ↑ general and central obesity; tuber DP: ↓ general and central obesity but ↑ underweight
Zhang et al., 2021 [54]	Three trajectories of a “southern” DP and a “modern” DP; four trajectories of a “meat” DP	Longitudinal	9299 (49.6% males)	Adults aged 18 years or older from the CHNS between 1991 and 2018	Overweight/obesity at each wave of survey collection	3 d food recalls	Highest initial score and a slight decrease trajectory of the meat DP: ↑ overweight/obesity; maintaining high southern DP and modern DP scores: ↓ overweight/obesity
Zou et al., 2017 [49]	“Cereal, animal, and plant food” DP; “high-protein food” DP; “plant food” DP; “poultry” DP; “beverage” DP	Cross sectional	1613 (46.8% males)	Adults from cities, townships, and residential villages in Zhejiang Province	BMI, overweight/obesity	24 h recall	Cereal, animal, and plant food DP and beverage DP: ↑ obesity

CHNS—China Health and Nutrition Survey; CI—confidence interval; DP—dietary pattern; FFQ—food frequency questionnaire; JIN—Jiangsu Nutrition Study; OR—odds ratio; PR—prevalence ratio; RRR—relative risk ratio; WC—waist circumference; “↑”—increase; “↓”—decrease.

## Supplementary Materials

The following are available online at https://www.mdpi.com/article/10.3390/nu14224911/s1. Table S1. Detailed summary of characteristics of included studies on dietary patterns and obesity or weight outcomes; Table S2: Quality assessment of included studies.
