# Supplementary material for: Dietary Patterns and Obesity in Chinese Adults: A Systematic Review and Meta-Analysis
Source: Nutrients. 2022 Nov 20;14(22):4911. doi: 10.3390/nu14224911 (PMC9698822; doi:10.3390/nu14224911)
Supplement: Supplementary file 1 [file nutrients-14-04911-s001.zip › nutrients-2030279-supplementary/Supplemental Table S1.pdf]

Supplementary Table S1. Detailed summary of characteristics of included studies on dietary patterns and obesity or weight outcomes

Number	Pattern	Study Type	Participants	Population	Outcome	Adjusted variables	Food Measurements	Findings
Cao et al. 2020 [50]	Trajectory of the "Traditional" DP with rice, meat, and vegetables; "modern" DP with fast food, milk, and deep-fried food over the survey collection period	Longitudinal	6,943 (48.4% males)	Adults aged over 20 years in the China Health and Nutrition Survey (CHNS) between 1991 and 2009	BMI, overweight/obesity in 2009	Age, gender, education, income, urbanization, smoking, alcohol drinking, physical activity	3-day 24 hr recall	High and stable traditional DP trajectory: lower BMI (β = -1.14; 95% CI: -1.41, -0.87); high and rapid increase in the modern DP: higher BMI (β = 0.74; 95% CI: 0.34, 1.15).
Cempaka et al. 2019 [32]	"Dysregulated iron metabolism related" DP with high intake of deep-fried food, processed meats, chicken, pork, eating out, coffee, and animal fat/skin but low intake of steamed/boiled/raw foods and dairy products	Cross-sectional	208 (50.4% males)	Taiwanese adults aged 20–65 years	Central obesity, fat mass	Age, gender, and body-mass index	FFQ	Central obesity (OR = 1.57; 95% CI: 1.05, 2.34); Visceral fat mass (%) (β = 0.23; 95% CI: 0.011,0.453)
Chan et al.2012 [33]	"Vegetables–fruit" DP with vegetables, fruits, legumes, soya and soya products; "snacks–drinks–milk products" DP with condiments, coffee, fast food, French fries, potato chips, nuts, and milk products; "meat–fish" DP with dim sum, red and processed meats, poultry, fish and seafood	Cross-sectional	3,707 (52.5% males)	Adults aged 65 years and above living in Hong Kong	BMI, WC, HC, waist-to-hip ratio	Age, PASE, daily energy intake, education level, Hong Kong ladder, community ladder, alcohol use, smoking status	FFQ	meat–fish DP: higher BMI (*β* = 0·19, 95 % CI: 0·06, 0·33), waist-to-hip ratio (*β* = 0·004, 95 % CI: 0·002, 0·007), and WC (*β* = 0·57, 95 % CI: 0·18, 0·97) in men, and higher BMI (*β* = 0·40, 95 % CI: 0·22, 0·57), WC (*β* = 0·87, 95 % CI: 0·39, 1·36) and HC (*β* = 0·61, 95 % CI: 0·26, 0·96) in women; "snacks-drinks-milk products" DP: lower waist-to-hip ratio (β = -0·004, 95 % CI: -0·007, -0·001) in men

Chen et al.2021 [34]	“Lacto-ovo-vegetarian” DP with tubers, vegetables, eggs, dairy products; “meat-fish” DP with meat, fish and seafood; junk food DP with fried food, desserts, beverages	Cross sectional	3,795 (37.2% males)	Community-dwelling older adults aged over 60 years in Shenyang, Liaoning province	Sarcopenic obesity, WC, obesity	The two other dietary patterns, age, sex, education level, cohabitation status, marital status, history of falls in the past year, exercise status, hemoglobin, alanine aminotransferase, triacylglycerols, high-density lipoprotein cholesterol, low-density lipoprotein cholesterol, fasting blood glucose, free triiodothyronine, and FT4	FFQ	Lacto-ovo-vegetarian DP: lower risk of sarcopenic obesity (OR = 0.79, 95% CI: 0.65, 0.97) ; no association with WC
Li et al. 2017 [51]	Mean cumulative DP scores during 1991-2011 of “Traditional” DP with rice, meat, and vegetables; “modern” DP with fast food, milk, and deep-fried food	Longitudinal	9,499 (48% males)	CHNS between 1991 and 2011	Overweight/obesity (BMI> 25 kg/m²); abdominal obesity in 2009	Urbanization, education, income, smoking, physical activity, alcohol	3-day 24 hr recall	Traditional DP: lower risk of obesity (β = -0.10, 95 % CI: 0·013, 0·07) and abdominal obesity (β = -0.13, 95 % CI: -0·16, -0·10); Modern DP: higher risk of obesity (β = 0.16, 95 % CI: 0·13, 0·19) and abdominal obesity (β = 0.12, 95 % CI: 0·09, 0.15)
Li et al. 2022 [35]	"Animal-based and processed food" DP with refined rice and noodle, meat and aquatic products; "traditional food" DP with whole grains, vegetables, and legumes; and "ovo-lacto vegetarian food" DP with dairy, fruits, and eggs	Cross sectional	1,136 (100% males)	Males aged over 65 years in Sichuan province	BMI, overweight/obesity, WC	Age, living status, education level, smoking status, alcohol consumption, and total energy intake	FFQ	Traditional DP: lower risk of overweight/obesity (OR= 0.51, 95% CI: 0.36, 0.72); animal-based and processed food DP: higher risk of overweight/obesity (OR = 3.25, 95% CI: 1.94, 5.46)

Meng et al. 2014 [36]	"Western food" DP with meat, processed meat, poultry, and beverages; "high protein and calcium" DP with soy products, dairy, and eggs; "fruits and snacks" DP with fruits, seafood, eggs, and snacks; "staple food and vegetables" DP with staple food and vegetables	Cross sectional	1,535 (47.4% males)	Adults aged > or = 18 years old in Shanghai	Overweight/obesity	Age, gender and degree of education	FFQ	Staple food and vegetables DP: higher risk of obesity (OR = 2.67)
Mu et al. 2014 [37]	"Western food" DP with hamburger and fried food, nuts, snack food, cola, coffee, sugars; "high protein and calcium" DP with pork, mutton, beef, poultry meat, animal liver; "Calcium food" pattern with milk and dairy products, beans and bean products, fresh fruit, eggs, fish and shrimp, kelp laver, and sea fish; "Chinese traditional" pattern with grains, fresh vegetables and fruits, pork	Cross sectional	1,319 (38.7% males)	College freshmen aged 16-20 years in Anhui province	Overweight/obesity	Sex, physical activity, economic status, passive smoking, drinking, calcium supplements	FFQ	Western food DP: higher risk of overweight/obesity (OR = 2.00, 95% CI: 1.24-3.22); traditional DP: lower risk of overweight/obesity (OR = 0.65, 95% CI: 0.53, 0.80).
Muga et al.2017 [38]	"Vegetable-fruit" DP rich in fruits and vegetables, meat-processed dietary pattern rich in meat and processed food	Cross sectional	62,965 (52% males)	Taiwanese adults aged over 40 years in the Mei Jau health screening institutions' database collected from 2001 and 2010	Overweight/obesity	Age, education level, marital status, smoking, drinking, physical activity, CVD, SBP, DBP	FFQ	Vegetable-fruit DP: lower risk of being overweight (OR = 0.91; 95% CI: 0.85, 0.97) or obese (OR = 0.85; 95% CI: 0.78, 0.92); meat and processed DP: higher risk of being overweight (OR = 1.50; 95% CI: 1.40, 1.59) or obese (OR = 1.94; 95% CI, 1.79, 2.10)

Shi et al.2008 [39]	“Traditional” DP with rice, fresh vegetables, pork and wheat flour; “vegetable-rich” DP with whole grains, fruits, root vegetables, fresh and pickled vegetables, milk, eggs and fish; “macho” DP with meat and alcohol; “sweet tooth” DP with drinks and cake	Cross sectional	2,849 (45.9% males)	Adults aged over 20 years in the Jiangsu Nutrition Study (JIN)	Overweight/obesity	Age, income, education, occupation, active commuting, smoking, drinking, energy intake	FFQ	Vegetable-rich DP: higher risk of general obesity (PR = 2.06, 95% CI: 1.46, 2.89)
Shi et al.2011 [52]	“Traditional” DP with rice, fresh vegetables, pork and wheat flour; vegetable-rich DP; “macho” DP with meat and alcohol; “sweet tooth” DP with drinks and cake assessed at baseline	Longitudinal	1,231 (41.4% males)	JIN 2002-2007	Weight gain during the survey period	Age, sex, education, occupation, active commuting, leisure-time physical activity, smoking and alcohol drinking, energy intake	FFQ	Traditional DP: lower weight gain (β = - 2·18, 95 % CI: - 2·91, - 1·45); Vegetable rich DP: greater weight gain (β = 1·00, 95 % CI: 0·25, 1·74)
Shu et al. 2015 [40]	“Animal food” DP with rice, mushrooms, red meat, fish, seafood, fat/oils; “traditional Chinese” DP with rice, coarse grains, steamed bun/noodles, tubers, fresh fruits and vegetables, fish and shrimp, and Miscellaneous bean, tea; “western fast-food” DP with fast foods, snacks, chocolates, coffee and drinks; “high-salt” DP with pickled vegetables, processed and cooked meat, bacon and salted fish, and bean sauce	Cross sectional	2,560 (53% males)	Adults aged 45-60 years from Zhejiang province	BMI, WC, waist to hip ratio (WHR), abdominal obesity	Age, smoking status, economic income, educational level, physical activity level, and total energy intake	FFQ	Animal DP: higher BMI (r = 0.08, 0.14, for men and women respectively) and WC (r = 0.10, 0.13), higher risk of abdominal obesity (OR=1.67, 95% CI: 1.19, 2.34); traditional Chinese DP: lower BMI (r = -0.05, -0.12) and WC (r = -0.07, -0.11), lower risk of abdominal obesity (OR = 0.63; 95% CI: 0.44, 0.90); Western DP not associated with obesity

Wang et al. 2021 [41]	"Traditional" DP with poultry, light-colored vegetables, red meat and its products, cereals and tubers products, condiment, oils and dark-colored vegetables; "fruit-egg" DP with fruit, whole grains, pickled vegetables and eggs and eggs products; "nut-wine" DP with nut, wine and pastry snacks	Cross sectional	1,739 (46.2% males)	Adult participants aged over 18 years in Jiangsu province	Overweight/obesity	Energy intake, age group, education level, job, smoking and income status	FFQ	Traditional DP: higher risk of overweight and obesity in men (OR = 1.95; 95% CI: 1.26, 3.04; β =0.12) but not in women
Xu et al. 2015 [42]	"Traditional" DP with high intake of rice, pork and vegetables; "modern" DP with high intake of fruit, fast food, and processed meat	Cross sectional	2,745 (47.4 % males)	2009 CHNS participants aged ≥ 60 years	Obesity	Age, marital status, work status, education level, smoking, physical activity, the other DP, and energy	3-d food recalls	Traditional DP: lower risk of overweight (RRR = 0.64, 95% CI: 0.45, 0.92) and general obesity (RRR = 0.66, 95% CI: 0.34, 1.27); Modern DP: higher risk of central obesity (PR=1.42, 95% CI:1.10, 1.67) in men, as well as lower risk of underweight (PR = 0.24, 95% CI: 0.09; 0.63) in women
Xu et al. 2016 [53]	"Traditional" and "modern" DP as above in four survey years	Longitudinal	6,348 (47.3% males)	CHNS 2004-2011 waves of participants aged ≥ 60 years	BMI, weight and WC changes in four survey years	Age, urbanization, gender, marital status, work status, education level, smoking, physical activity, the other DP and energy	3-d food recalls	Traditional DP: greater BMI decrease (β = -0.23, 95 % CI: -0.44, -0.02), weight decrease (β = -0.90, 95 % CI: -1.42, -0.37); and WC decrease (β = -1.57, 95 % CI: -2.32; -0.83). Modern DP: BMI increase (β = 0.29, 95 % CI: 0.12; 0.47); weight increase (β = 1.02, 95 % CI: 0.58; 1.46); WC increase (β = 1.44, 95 % CI: 0.78, 2.10)

Ye et al. 2018 [43]	"Healthy traditional" DP with vegetables, rice and products, fish and shrimp; "animal and plant protein" DP with poultry meat, dry beans, other soyabean products and livestock meat; "condiments" DP with soya sauce, sugar, vinegar and salt; "fruits, eggs, and juice" DP with fruits, eggs, soyabean milk, juice, nuts, cola and sweets; "alcohol, milk and tea" DP with beer, liquor, dairy products, wine and tea.	Cross sectional	3,376 (41.4% males)	Adult participants aged over 35 years in a community-based nutrition and health survey in 2007 in urban areas of Nanjing	Abdominal obesity	Age, sex, education level, family annual income, smoking status and alcohol consumption	FFQ	Healthy traditional DP: lower risk of abdominal obesity (OR = 0.52; 95 % CI: 0.41, 0.67).
Yu et al. 2015 [44]	“Traditional south” DP with high rice and low wheat intakes; “traditional north” DP with high wheat but low rice, meat, poultry, fish and fresh fruit intakes; “Western” DP with fresh fruit and protein products	Cross sectional	474,192 (59% males)	Adults aged 30–79 years from the China Kadoorie Biobank	BMI, WC, general obesity, central obesity	Age, sex, study area, marital status, education level, household income, alcohol consumption, smoking status, and physical activity	FFQ	Traditional southern DP: lower risk; Traditional north DP: general obesity: (PR = 1.05, 95% CI: 1.02, 1.09) and central obesity (PR = 1.17, 95% CI, 1.15, 1.18); Western DP: general obesity: (PR = 1.06, 95% CI: 1.03, 1.08) and central obesity (PR = 1.07, 95% CI:1.06, 1.08)
Zhang et al. 2012 [45]	“Vegetable” DP with vegetables, fruits, tubers, and starches; “sweets and fats” DP with sugars and preserves, condiments, and oil; “legume” DP with beancurds, legumes, eggs, organ meat, and course cereals; and “poultry, beef and mutton” DP with poultry, beef and mutton, fruits, tubers and starches, coarse cereals, organ meat, and condiments	Cross sectional	556 (50.5% males)	Newlywed couples aged under 35 years in Shanghai	Weight gain	Age, sex, smoking and alcohol consumption, education, physical activity, staying up late, living with parents and body weight before marriage	FFQ	Sweets and fats DP: more weight gain after marriage (β = 2·94; 95 % CI: 0·75, 5·15; P = 0.01) in men; poultry, beef and mutton DP: less weight gain after marriage (β = −1·21; 95 % CI: −2·32, −0·11; P = 0·03)

Zhang et al. 2014 [46]	"Animal food" DP with meat, snacks, and sugar sweetened beverages; "plant food" DP with non-starchy vegetables, mushrooms, beans, and starchy vegetables; "seafood" DP with fish, seafood, fruits, eggs, snacks, and dairy	Cross sectional	2,116 (46.6% males)	Adults aged over 18 years in the "China National Nutrition and Health Status Monitoring" cohort	Abdominal obesity	Age, sex, marital status, education, family income, smoking, alcohol drinking, family history of chronic diseases	FFQ	Increased risks of abdominal obesity with animal food pattern (OR = 1.88), inverse association with seafood pattern (OR = 0.83)
Zhang et al. 2015 [47]	"Traditional south" DP with rice, vegetables, and pork; "traditional north" DP with wheat, other cereals, and tubers; "snack" DP with fruits, eggs, milk, and nuts; and "high protein" DP with fungi, algea, legume, seafoods, pork	Cross sectional	2,363 (100% females)	Women aged 18-44 years in the 2011 CHNS	obesity, BMI, WC	Age, education, living area, smoking status, drinking status, physical activity, annual household incomes per family member and total energy intake	3-d food recalls	Traditional south DP: lower risk of general obesity (OR = 0.48; 95% CI: 0.29, 0.78) and abdominal obesity (OR = 0.64; 95% CI: 0.46, 0.90); traditional north DP: greater risk of general obesity (OR = 2.28; 95% CI: 1.38, 3.74) and abdominal obesity (OR = 2.32; 95% CI: 1.66, 3.24)
Zhang et al. 2016 [48]	"Modern" DP with vegetables, milk, eggs, meat, wheat, beans, and fast food; "traditional" DP with wheat, cake, and oil; "tuber" DP with tubers, fruits, and cakes	Cross sectional	1,604 (41.4% males)	Adults aged 18-80 years in the 2010–2012 National Nutrition Survey in Yunnan province	Obesity, BMI, WC	Age, sex, energy intake, physical activity, smoke, and drink	3-d food recalls	Modern DP: higher risk of general and central obesity (OR = 1.95, 95% CI:1.15, 3.48; OR = 2.01, 95% CI: 1.37, 2.93); tuber pattern: lower risk of general and central obesity (OR = 0.34, 95% CI: 0.15, 0.61; OR = 0.64, 95% CI: 0.43, 0.95) but higher risk of underweight (OR = 2.57, 95% CI: 1.20, 6.45)
Zhang et al. 2021 [54]	Three trajectories of a "southern" DP with rice, vegetables, and pork and a "modern" DP with fruits, dairy, and processed food; four trajectories of a "meat" DP with organ meats,	Longitudinal	9,299 (49.6% males)	Adults aged 18 years or older from the CHNS between 1991 and 2018	Overweight/obesity at each wave of survey collection	Baseline age, gender, living areas, individual income, education level, physical activity, smoking status, alcohol intake, energy intake, baseline BMI	3-d food recalls	Highest initial score and a slight decrease trajectory of the meat DP: higher risk of overweight/obesity (OR = 1.63; 95% CI: 1.04, 2.54); maintaining high Southern DP and modern DP scores: lower

	poultry, pork and other livestock meat						risk of overweight/obesity (OR = 0.64; 95% CI: 0.51, 0.81; OR = 0.71; 95% CI: 0.54, 0.91), (OR = 0.76; 95% CI: 0.63, 0.91; OR = 0.64; 95% CI: 0.44, 0.90)
Zou et al. 2017 [49]	"Cereal, animal, and plant food" DP with correlations with pork, vegetables, cereals, fish and shellfish; "high protein food" DP with milk and dairy products, eggs, fish, and shellfish; "plant food" DP with beans, fruits, and nuts; "poultry" DP with chicken and duck; "beverage" DP with carbonated beverage and fruit drink	Cross sectional	1,613 (46.8% males)	Adults from cities, townships, and residential villages in Zhejiang Province	BMI, overweight/obesity	Gender, age, education, smoking, total energy intake, household income	24-hr recall	Cereal, animal, and plant food as well as beverage DP: higher risk of obesity (OR = 2.924, 3.257; 95% CI: 1.147, 7.463; 1.372, 7.692)

CHNS—China Health and Nutrition Survey; CI—confidence interval; DP—dietary pattern; FFQ—food frequency questionnaire; JIN—Jiangsu Nutrition Study; OR—odds ratio; PR—prevalence ratio; RRR—relative risk ratio; WC—waist circumference. ; "↑"—increase; "↓"—decrease
