# Supplementary material for: Dietary Patterns and Obesity in Chinese Adults: A Systematic Review and Meta-Analysis
Source: Nutrients. 2022 Nov 20;14(22):4911. doi: 10.3390/nu14224911 (PMC9698822; doi:10.3390/nu14224911)
Supplement: Supplementary file 1 [file nutrients-14-04911-s001.zip › nutrients-2030279-supplementary/Supplementary Table S2.pdf]

Supplementary Table S2. Quality assessment of included studies.

Criteria	Cao et al.	Cem-paka et al.	Chan et al.	Chen et al.	Li et al. 2017	Li et al. 2022	Meng et al.	Mu et al.	Muga et al.	Shi et al. 2008	Shi et al. 2011	Wang et al.	Xu et al. 2015	Xu et al. 2016	Ye et al.	Yu et al.	Zhang et al. 2021	Zhang et al, 2015	Zhang et al. 2016	Zhang et al. 2014	Zhang et al. 2011	Zou et al.
1. Was the research question or objective in this paper clearly stated?	1	1	1	1	1	1	1	1	1	1	1	1	1	1	1	1	1	1	1	1	1	1
2. Was the study population clearly specified and defined?	1	1	1	1	1	1	1	1	1	1	1	1	1	1	1	1	1	1	1	1	1	1
3. Was the participation rate of eligible persons at least 50%?	1	1	1	1	1	1	1	1	1	1	1	1	1	1	1	1	1	1	1	1	1	1
4. Were all the subjects selected or recruited from the same or similar populations (including the same time period)? Were inclusion and exclusion criteria for being in the study prespecified and applied uniformly to all participants?	1	1	1	1	1	1	1	1	1	1	1	1	1	1	1	1	1	1	1	1	1	1
5. Was a sample size justification, power description, or variance and effect estimates provided?	1	1	1	1	1	1	1	1	1	1	1	1	1	1	1	1	1	1	1	1	1	1
6. For the analyses in this paper, were the exposure(s) of interest measured prior to the outcome(s) being measured?	1	0	0	0	1	0	0	0	0	0	1	0	0	1	0	0	1	0	0	0	0	0
7. Was the timeframe sufficient so that one could reasonably expect to see an association between exposure and outcome if it existed?	1	0	0	0	1	0	0	0	0	0	1	0	0	1	0	0	1	0	0	0	0	0
8. For exposures that can vary in amount or level, did the study examine different levels of the exposure as related to the outcome (e.g., categories of exposure, or exposure measured as continuous variable)?	1	0	0	0	1	1	1	1	1	1	1	1	1	1	0	0	1	1	1	1	1	1
9. Were the exposure measures (independent variables) clearly defined, valid, reliable, and implemented consistently across all study participants?	1	1	1	1	1	1	1	1	1	1	1	1	1	1	1	1	1	1	1	1	1	1
10. Was the exposure(s) assessed more than once over time?	1	0	0	0	1	0	0	0	0	0	0	0	0	1	0	0	1	0	0	0	0	0
11. Were the outcome measures (dependent variables) clearly defined, valid, reliable, and implemented consistently across all study participants?	1	1	1	1	1	1	1	1	1	1	1	1	1	1	1	1	1	1	1	1	1	1
12. Were the outcome assessors blinded to the exposure status of participants?	1	1	1	1	1	1	1	1	1	1	1	1	1	1	1	1	1	1	1	1	1	1
13. Was loss to follow-up after baseline 20% or less?	1	0	0	0	1	0	0	0	0	0	1	0	0	1	0	0	1	0	0	0	0	0
14. Were key potential confounding variables measured and adjusted statistically for their impact on the relationship between exposure(s) and outcome(s)?	1	1	1	1	1	1	1	1	1	1	1	1	1	1	1	1	1	1	1	1	1	1
Total score	14	9	9	9	14	10	10	10	10	10	13	10	10	14	9	9	14	10	10	10	10	10
Risk	Low	Me-dium	Me-dium	Me-dium	Low	Me-dium	Me-dium	Me-dium	Me-dium	Me-dium	Low	Me-dium	Me-dium	Low	Me-dium	Me-dium	Low	Me-dium	Me-dium	Me-dium	Me-dium	Me-dium
